# Comparing the Efficiency of Two Treatment Methods of Hydrocephalus: Shunt Implantation and Endoscopic Third Ventriculostomy

**DOI:** 10.32598/bcn.9.10.285

**Published:** 2019-05-01

**Authors:** Seifollah Gholampour, Mehrnoush Bahmani, Azadeh Shariati

**Affiliations:** 1. Department of Biomedical Engineering, Faculty of Electrical & Computer Engineering, Tehran North Branch, Islamic Azad University, Tehran, Iran.

**Keywords:** Cerebrospinal Fluid, Intracranial Pressure, Intracranial compliance, Survival curve, Reynolds number, CSF velocity

## Abstract

**Introduction:**

Hydrocephalus is one of the most common diseases in children, and its treatment requires brain operation. However, the pathophysiology of the disease is very complicated and still unknown.

**Methods:**

Endoscopic Third Ventriculostomy (ETV) and Ventriculoperitoneal Shunt (VPS) implantation are among the common treatments of hydrocephalus. In this study, Cerebrospinal Fluid (CSF) hydrodynamic parameters and efficiency of the treatment methods were compared with numerical simulation and clinical follow-up of the treated patients.

**Results:**

Studies have shown that in patients under 19 years of age suffering from hydrocephalus related to a Posterior Fossa Brain Tumor (PFBT), the cumulative failure rate was 21% and 29% in ETV and VPS operation, respectively. At first, the ETV survival curve shows a sharp decrease and after two months it gets fixed while VPS curve makes a gradual decrease and reaches to a level lower than ETV curve after 5.7 months. Post-operative complications in ETV and VPS methods are 17% and 31%, respectively. In infants younger than 12 months with hydrocephalus due to congenital Aqueduct Stenosis (AS), and also in the elderly patients suffering from Normal Pressure Hydrocephalus (NPH), ETV is a better treatment option. Computer simulations show that the maximum CSF pressure is the most reliable hydrodynamic index for the evaluation of the treatment efficacy in these patients. After treatment by ETV and shunt methods, CSF pressure decreases about 9 and 5.3 times, respectively and 2.5 years after shunt implantation, this number returns to normal range.

**Conclusion:**

In infants with hydrocephalus, initial treatment by ETV was more reasonable than implanting the shunt. In adult with hydrocephalus, the initial failure in ETV occurred sooner compared to shunt therapy; however, ETV was more efficient.

## Highlights

- Although in adult hydrocephalus, the effectiveness of shunt implantation is better in the short term, Endoscopic Third Ventriculostomy (ETV) is a more appropriate option in the long term.
- Cerbrospinal Fluid (CSF) volume and more importantly CSF pressure are the most effective hydrodynamic parameters in evaluating the treatment methods of hydrocephalus.
- After treatment by the ETV and shunt methods, CSF pressure decreases about 9 and 5.3 times, respectively.
- ETV is a potentially safer option for hydrocephalus in patients with osteoporosis.

## Plain Language Summary

The imbalance between CSF production and absorption or CSF path obstruction results in hydrocephalus. It is one of the most common diseases in children. Endoscopic Third Ventriculostomy (ETV) and Ventriculoperitoneal Shunt (VPS) implantation are among the common treatment methods of hydrocephalus. However, the pathophysiology of the disease is still unknown. ETV is a better treatment option in infants younger than 12 months with hydrocephalus due to congenital Aqueduct Stenosis (AS) and also elderly patients suffering from Normal Pressure Hydrocephalus (NPH). In adult hydrocephalus, the long-term effectiveness of ETV is better. Volume and especially pressure were the most effective parameters in evaluating the treatment methods of hydrocephalus. After treatment by the ETV and shunt methods, CSF pressure decreases about 9 and 5.3 times, respectively and 2.5 years after shunt implantation, this number returns to the normal range.

## 1. Introduction

Hydrocephalus is one of the most common diseases in children (Karimy et al., 2016; Muir, Wang, & Warf, 2016). In developed countries, congenital hydrocephalus prevalence is 0.5–1 per 1000 live-born infants. Whereas, the prevalence of acquired hydrocephalus is 3 to 5 per 1000 live-born infants (Wiswell, Tuttle, Northam, & Simonds, 1990; Chi, Fullerton, & Gupta, 2005; Munch et al., 2012; Tully & Dobyns, 2014). The prevalence of idiopathic Normal Pressure Hydrocephalus (iNPH) has been reported to be 1.8 to 2.2, respectively per 100000 and 1000000 people (Gallia, Rigamonti, & Williams, 2006).

To properly understand the hydrocephalus, it is necessary to initially discuss the production and absorption of Cerebrospinal Fluid (CSF) and its pathway. CSF is mainly produced by choroid plexus in the lateral, third and fourth ventricles (Wise & Schlosser, 2007; Brinker, Stopa, Morrison, & Klinge, 2014). Ependymal cells and capillaries also play minor roles in the secretion of CSF (Kagerbauer et al., 2013). Through arachnoid granulations, CSF will be drained into venous sinuses and then to the lymphatic system via Virchow-Robin spaces. Next, it will be mostly drained into craniospinal nerves through perineural space; while some part of it will be drained into the spinal cord (Brinker et al., 2014; Ducros & Biousse, 2015).

CSF circulates within cerebral ventricles (laterals, the third and fourth ventricles and aqueducts) and cerebrospinal Subarachnoid Space (SAS) (Sakka, Coll, & Chazal, 2011). In general, CSF volume is about 160 mL that 25% of this volume is circulating within ventricles and 75% in spinal and subarachnoid cortical spaces (Bateman, Stevens, & Stimpson, 2009). The Mean±SD production rate of CSF is equal to 0.34±0.13 mL/min; the average CSF absorption rate in the spinal cord is 0.17 mL/min (Edsbagge, Tisell, Jacobsson, & Wikkelso, 2004). It should be mentioned that under normal physiological condition, CSF circulation has fixed inflow and pulsing (Davis & Cushing 1925; Taketomo & Saito, 1965; Milhorat, 1975).

The most important hydrodynamic parameter indicating the incidence of hydrocephalus is the CSF pressure (Gholampour, Fatouraee, Seddighi, & Yazdani, 2014; Fatouraee, Gholampour, & Seddighi, 2015; Hajirayat, Gholampour, Seddighi, & Fatouraee, 2016; Gholampour, Hajirayat, Erfanian, Zali, Shakouri, 2017; Gholampour, Fatouraee, Seddighi, & Seddighi, 2017a; Gholampour, Fatouraee, Seddighi, & Seddighi, 2017b; Gholampour, 2018; Gholampour & Taher, 2018).

Intracranial Pressure (ICP) refers to the numerical values of CSF pressure in upper convexity of the brain in SAS. It should be noted that ICP wave differs from the Arterial Blood Pressure (ABP) wave (Schmidt et al., 2018). ICP values in normal infants younger than one year, children, and adults are respectively 3–4 mm Hg, 11 mm Hg, and 10–15 mm Hg (Ekstedt, 1978; Malm, Jacobsson, Birgander, & Eklund, 2011; Sakka et al., 2011; Lawley et al., 2015). Biological fluid flow such as CSF for healthy subjects and also hydrocephalus patients have been described through Navier-Stokes and Arbitrary Lagrangian-Eulerian (ALE) equations (Ma, Liu, Zu, & Tang, 2012; Gholampour et al., 2017a), using Computational Fluid Dynamics (CFD) solution methods and Fluid-Structure Interaction (FSI) simulation.

Many CSF circulation parameters such as CSF flow velocity and flow rate diagrams for patients with hydrocephalus and healthy subjects are measured via Cine phase contrast Magnetic Resonance Imaging (Cine PC-MRI). Meanwhile, these equipment are more useful to understand the patients’ pathophysiology (Linninger et al., 2007; Akutsu et al., 2018). ICP may be measured by an invasive method, ICP monitoring, and or by noninvasive computer simulations as CFD and FSI (Eide, Holm, & Sorteberg, 2012; Gholampour et al., 2014; Fatouraee et al., 2015).

## 2. Hydrocephalus

The imbalance between production and absorption of CSF or obstruction of CSF flow path results in hydrocephalus. This causes ventricular dilatation and increases ICP (Langner et al., 2017). It is almost one century ago that Dandy performed the first empirical studies on hydrocephalus (Greitz, 2004).

### 2.1. Various types of hydrocephalus

Dandy and Blackfan categorized hydrocephalus in three groups of Non-Communicating Hydrocephalus (NCH), Communicating Hydrocephalus (CH), and Normal Pressure Hydrocephalus (NPH) (Dandy & Blackfan, 1914; Eide & Pripp, 2016). There are numerous definitions for these three groups. However, the most common definitions for these groups are as follows:

#### 2.1.1. Communicating hydrocephalus

CSF circulation path in Subarachnoid Space is obstructed, whereas CSF is still circulating between brain ventricles (Hakim, & Adams, 1965; Tasiou, Brotis, Esposito, & Paterakis, 2016). Communicating Hydrocephalus (CH) results from obstruction in basic cisterns level or arachnoid villi. It is also called extra-ventricular obstructive hydrocephalus (Rekate, 2009).

#### 2.1.2. Non-Communicating Hydrocephalus

An obstruction or abnormality exists in CSF flow within the ventricular system. Usually, the Sylvius aqueduct, a connection between the third and fourth ventricles of the brain, is blocked, resulting in obstruction of CSF flow through ventricles (Sæhle & Eide, 2015; Eide & Pripp, 2016). NCH is mostly called obstructive hydrocephalus and is described as intraventricular CSF flow obstruction (Maller & Gray, 2016).

#### 2.1.3. Normal pressure hydrocephalus

It is a chronic disorder resulted from interrupted CSF absorption or flow (Kang et al., 2018), when CSF volume increases in the lateral, third and fourth ventricles, with no considerable increase in ICP. The pathophysiology is still unknown (Bateman, 2000).

In another classification based on hydrodynamics, hydrocephalus is divided into two main types of acute and chronic. Conventionally, acute hydrocephalus is caused by intraventricular obstruction. Chronic hydrocephalus creates arterial pulsations and increase of capillary pulsations, because of reduction of intracranial compliance (ΔV/ΔP) (Greitz, 2004). Acute hydrocephalus is diagnosed via compression of venous outflow by dilated capacitance arteries. Chronic hydrocephalus is diagnosed through compression of capacitance vessels and reduction of compliance (Greitz, 2004). In the acute phase, ICP reduction is related to fluid draining from obstructed ventricles. In the chronic phase, intracranial compliance increase is related to the increase of capacitance vessels flow (Greitz, 2004).

Dandy-Walker Syndrome (DWS) is also a type of congenital anomaly with a lack of formation of cerebellar vermis or it being small, cystic fourth ventricle, and large posterior fossa as its symptoms. The term Chiari Malformation (CM) alludes to the caudal displacement of the cerebellar tonsils through the foramen magnum. DWS and CM may occur with or without hydrocephalus (Khoshnevisan, Sistani Allah Abadi, & Abdollahzadeh, 2012; Gholampour, 2018).

### 2.2. Hydrocephalus diagnosis and treatment methods

Hydrocephalus is mainly diagnosed clinically (Tasiou et al., 2016). However, Computed Tomography (CT) and Magnetic Resonance Imaging (MRI) play an important role in the diagnosis of hydrocephalus (Maller & Gray, 2016). Shunt implantation, ETV, and posterior fossa decompression are the prevalent methods for the treatment of hydrocephalus. In those cases accompanied by DWS or CM, these three methods could also be used for treatment (Greitz, 2004; Gholampour & Taher, 2018). The effectiveness of hydrocephalus treatment method also depends on the causes of the disease (Khoshnevisan et al., 2012).

#### 2.2.1. Shunt implantation

Shunt implantation treatment is an invasive method of treatment. A lateral perforation will be created in the brain and a catheter will be placed in one of the lateral ventricles (Thompson, 2017). Additional CSF will be drained under the skin through the catheter to the peritoneal cavity, pleural cavity, lung, or right atrium of the heart (Wallace, McConathy, Menias, Bhalla, Wippold, 2014; Thompson, 2017). In general, shunt implantation is the first choice of treatment for those suffering from NPH (Kang et al., 2018). It should be mentioned that one of the shunt malfunction factors may be brain compliance reduction (Fukuhara, Luciano, Brant, & Klauscie, 2001).

**Figure 1.**
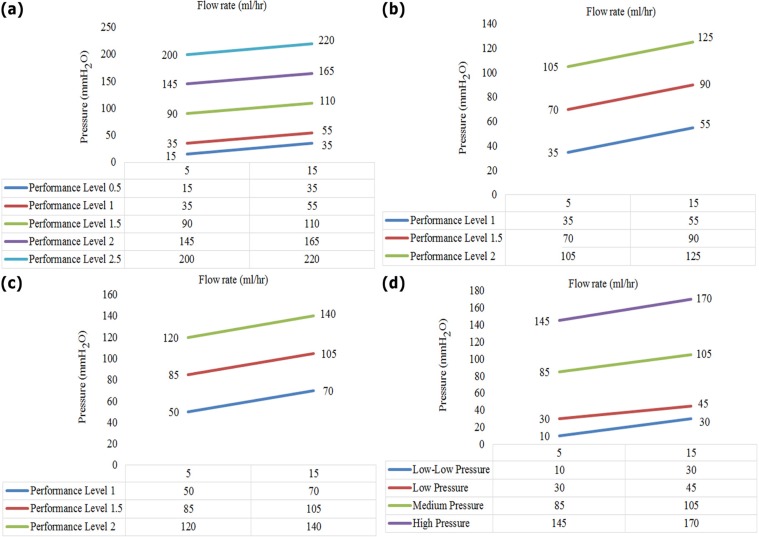
ICP ranges in different types of shunt A. Five pressure ranges in the model; B and C. Strata, pressure range in lying back and standing positions; D. Pressure range in CSF-flow control model

All shunts drain CSF from ventricle; however, their outputs are different. For example, if CSF is drained into the peritoneum, it is called ventriculo-peritoneal shunt (VPS) (Thompson, 2017); and if it is drained into the lung, it is called Ventriculo-Pleural Shunt (VPL). If CSF is drained into the atrium, it is called Ventriculo-Atrial (VA) shunt (Wallace et al., 2014). The most common concerns about hydrocephalus treatment are related to shunt implantation, while it has limited controlling options. In this regard, “smart shunt” may improve connection, feedback and telemetry controls. In this type of shunt, the physician may be informed of the patient’s status and performance of shunt through a smartphone or a sensor-based controller. Figure 1 shows the adjustment pressure and flow rate range for the shunts.

#### 2.2.2. Endoscopic Third Ventriculostomy

It is a minimally invasive method via the application of rigid and flexible endoscopy. In ETV method, the third ventricle floor is perforated to create a connection between ventricles and cisterns in SAS; which in turn results in ICP decrease (Harris, & McAllister, 2011; Spennato et al., 2011). The method is more useful in treating obstructive hydrocephalus or idiopathic hydrocephalus (Hakim & Adams, 1965; Fukushima, 1978; Tasiou et al., 2016). ETV or implanting a shunt in the patients suffering from chronic hydrocephalus do not mainly aim at absorbing CSF but to increase intracranial compliance (Greitz, 2004).

**Figure 2.**
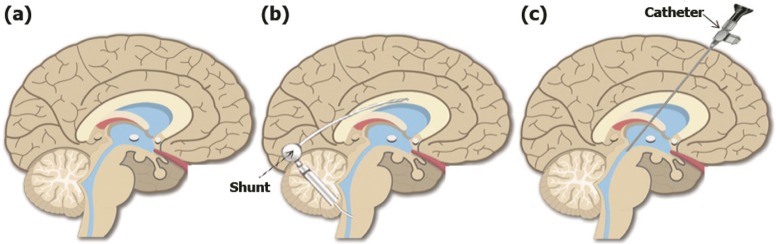
Brain in three states A. Healthy; B. Under shunt therapy; and C. Under ETV treatment

Figure 2 shows the brain in the three following situations: healthy status, treatment with a shunt, and treatment with ETV. ETV primarily aims at improving intracranial compliance status, through cerebral pulsation restoration and normalization of CSF flow (Fountas, Kapsalaki, Paterakis, Lee, Hadjigeorgiou, 2012; Tasiou et al., 2016). It should be noted that ETV is effective in patients suffering from Aqueduct Stenosis (AS) and is not much effective in patients with non-obstructive hydrocephalus (Bargalló et al., 2005).

#### 2.2.3. Posterior fossa decompression

In many cases, CM disorders are also accompanied by hydrocephalus. In many types of CM, physical damages in craniocervical junction disrupt CSF circulation in the fourth ventricle. In this case, posterior fossa decompression may be a good treatment option. The basic mechanism is to increase intracranial compliance, through posterior fossa decompression (Greitz, 2004; Eide & Pripp, 2016). The common challenges confronted with the “smart shunt” are humidity elimination and lack of recalibration after implantation (Lutz, Venkataraman, & Browd, 2013).

## 3. Comparing the effectiveness of VPS and ETV

Some in vivo and in vitro models have been proposed for simulation and or comparing VPS and ETV treatment methods. There have been fewer instances of complications such as malfunction or infection in ETV compared to shunt implantation; however, ETV is usually accompanied with hemorrhage and thalamus damage (Fountas et al., 2012; Tasiou et al., 2016). Also, studies have shown that ETV has relatively higher success and lower complications rate in iNPH patients. Minimally invasive ETV method is mainly effective in AS patients. However, there are several reports indicating that ETV has been effective, leading to improvement of ventricle size (Akutsu et al., 2018). It seems that ETV is a potentially safer option for hydrocephalus in patients with osteoporosis because the infection risk would be lower than that in shunt implantation. Shunt infection is one of the causes of death of patients with osteoporosis.

As VPS should also be removed, there are some points to be taken into consideration. Fukuhara et al. (2001) studied the effects of removing VPS on oxygen and brain compliance of the patients with chronic obstructive hydrocephalus. In their model, the changes in physiological parameters were evaluated in three untreated, shunt implanted, and shunt-removed phases and then brain compliance curve was measured in all phases.

**Figure 3.**
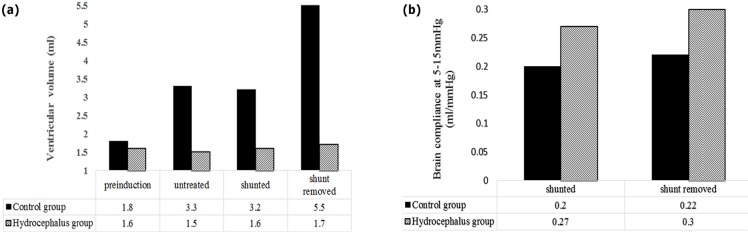
Comparison the ventricular volume and brain compliance a. Changes in the ventricle size before implanting shunt, when it is implanted, and after shunt removal; and b. Changes in brain compliance in low pressure before and after shunt removal.

In their study, adult dogs were used and 13 weeks after the operation, MRI was performed on dogs. The lateral and third ventricle size in addition to ICP in hydrocephalus dogs and the healthy group were compared. The results showed that, after shunt implantation in chronic obstructive hydrocephalus, the ventricle size and ICP decreased while O_2_ saturation and brain compliance increased and improvement was seen in response to hyperventilation in brain tissue (Figure 3).

Hyperventilation significantly reduced ICP and O_2_ saturation levels. It significantly reduced tissue O_2_ saturation, when the shunt was implanted (Fukuhara et al., 2001). The ventricle size in hydrocephalus patients also increased gradually and shunt implantation prevented this increase in size (Figure 3 a). Shunt removal reincreased the ventricle size.

### 3.1. Infant hydrocephalus

Regarding the prevalence of hydrocephalus in infants, evaluating the effectiveness of ETV and VPS in treating these patients is very important. However, there are different views on the effectiveness of these two treatment methods in infants.

Chowdhury et al. (2017) followed up for 4 years treatment of 1–2 years children suffering from AS-related hydrocephalus. The results showed that ETV treatment compared to shunt implantation is more appropriate for this group. Also, ETV has been proved to be a better option to treat congenital AS resulting in secondary NCH. Compared to post-hemorrhagic patients or post-infective hydrocephalus group, ETV had a better outcome in the aforementioned group.

**Figure 4.**
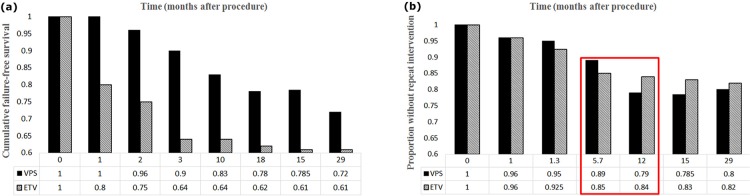
Results of survival curves measured by Kaplan-Meier method a) Comparison the results of survival curve for patients that treated with VPS and ETV; b) Comparison the proportion without repeat intervention in patients that treated with VPS and ETV

The results of Kulkarni et al. (2016) research on hydrocephalus infants showed that initial treatment through ETV was more reasonable than using shunts. The reason is that, in infants younger than 6 months, the failure rate is higher when treated with a shunt (Figure 4 a). In infants with hydrocephalus, depending on the type of treatment used either Endoscopic Third Ventriculostomy with Choroid Plexus Cauterization (ETV/CPC) or VPS treatment, various changes may occur in the craniometrics.

The results showed that in infants followed up 6 months after hydrocephalus treatment by ETV/CPC method, ventricle size remained unchanged, which is one of the success indexes in the treatment method (Dewan et al., 2018). Uche et al. Research showed that the mortality rate of those treated with ETV was lower than that of the VPS. Therefore, ETV is the first treatment option for children with Non-Communicating Non-Tumor Hydrocephalus (NCNTH), except in cases of severe macrocephaly (Uche, Okorie, Iloabachie, Amuta, & Uche, 2018). Li, Gui, and Zhang (2017) research showed that the failure rate and success rate of ETV and shunt implantation were similar one year after the treatment.

### 3.2. Pediatric hydrocephalus

In the following, we compare the effectiveness of these two methods of treating pediatric hydrocephalus. Limbrick et al. compared the pediatric hydrocephalus of 3–8 years old patients, in terms of treatment with shunt and ETV (Limbrick et al., 2014). General results of previous research showed that both methods were appropriate options to treat pediatric hydrocephalus patients (Limbrick, Baird, Klimo, Riva-Cambrin, Flannery, 2014; Tasiou et al., 2016).

### 3.3. Adult hydrocephalus

In adult hydrocephalus, the effectiveness of both ETV and VPS methods is of great importance. Dewan et al. studied the patients under 19 years suffering from hydrocephalus and Posterior Fossa Brain Tumor (PFBT), who were under treatment with VPS and ETV methods. Based on their results, the initial failure has occurred in ETV sooner than in shunt therapy. After 3 months, the failure rate in ETV has become lower than the shunt implantation. This shows the more lasting advantage of survival curves in ETV method (Dewan, Lim, Shannon, & Wellons, 2017).

After removing PFBT, the failure time in both ETV and VPS methods were computed, and survival curves were drawn via the Kaplan-Meier method. In medicine, the survival curve obtained through the Kaplan-Meier method is mostly used to measure the fraction of subjects living for a certain amount of time after treatment. In fact, the Kaplan-Meier survival curve is an observed function to estimate the survival function (Lacny et al.; Kaplan, & Meier, 1958).

In Figure 4 b, 5.7 months shows the first time VPS survival curve has reached to lower level than that of ETV method. Then, the obtained data were compared by the Wilcoxon rank-sum and the Chi-square tests. The results showed that failure in ETV occurred sooner than VPS method; however, ETV treatment was more lasting (about 12–29 months) (Figure 4 b, red rectangle). Thus, both ETV and VPS experience failure occurred, but as time passes VPS failure rate gets lower than that of ETV. Of course, these results were different from the results of the Kulkarni study (Figure 4 a), which was different for pediatric hydrocephalus after the 12^th^ month.

### 3.4. Elder hydrocephalus

Effectiveness of the two methods on the elderly people is also significant. However, a limited number of studies have been performed on this issue, including the one by Kang et al. (2018). Their results on 1 to 12 months follow-up of NPH patients (average age of 70) showed that ETV was more effective. Also, Tasiou et al. (2016) studied ETV treatment on iNPH patients.

## 4. Comparing changes of CSF hydrodynamics in ETV and VPS

**Figure 5.**
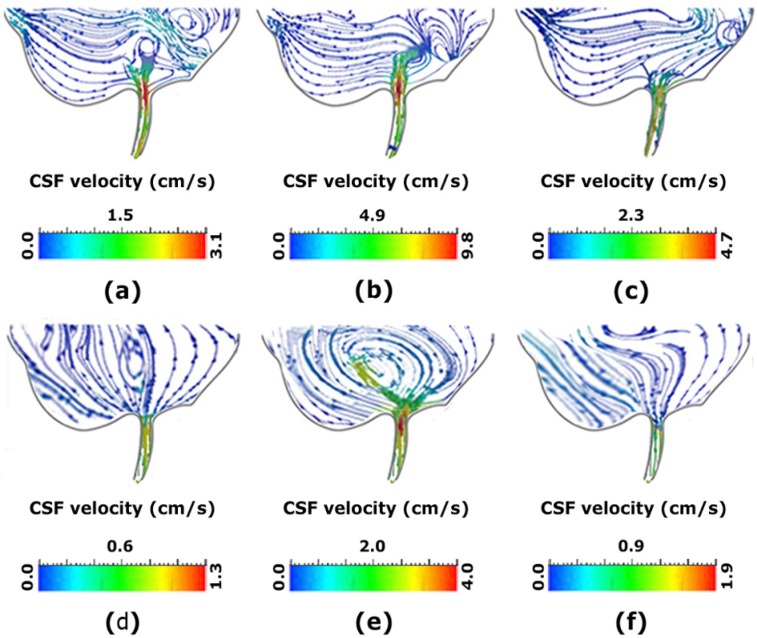
Comparison CSF velocity and vortex in samples a–c. Shows the vortex in the inferior section of the third ventricle of a patient with NCH before shunting; d–f. Shows the vortex in the inferior section of the third ventricle of a normal subject; b, e. Shows the vortex during the fill period; and c, f. Shows it during the flush period (Gholampour, 2018).

Many studies compared CSF hydrodynamic parameters in hydrocephalus patients and healthy subjects (Gholampour et al., 2014; Gholampour et al., 2017b; Gholampour, 2018). In some studies, brain compliance has also been examined in patients (Eide & Pripp, 2016; Gholampour, 2018). Meanwhile, in some other studies, shunt implantation and ETV have been simulated via computer. However, in none of these studies, a simultaneous and comprehensive comparison has been performed on the hydrodynamic parameters changes in the two treatment methods (Gholampour, Soleimani, Zalii, & Seddighi, 2016a; Hajirayat, Gholampour, Sharifi, & Bizari, 2017; Khademi, Mohammadi, Gholampour, & Fatouraee, 2016; Gholampour et al., 2016b; Gholampour, Fatouraee, Naderi, & Bagheri, 2019). Thus, we made such comparison separately in the continuation of this study. To evaluate the changes in CSF hydrodynamics, CSF flow conditions were simulated in healthy and patient charts (Figure 5).

Reynolds number is the index to determine the fluid laminar or turbulent flow (Hajirayat et al., 2016; Gholampour et al., 2017). In healthy subjects, Reynolds number is about 311 whereas, the mean number in AS patients is 409. After shunt operation, the value reduced to 329, indicating the parameter improvement after the operation (Gholampour et al., 2017a; Gholampour, 2018).

In all conditions, either before or after the treatment of patients and also in healthy subjects, the number of Reynolds remained within the laminar range. Gholampour studied and compared CSF hydrodynamics via FSI simulation in NCH patients before shunt implantation and 2.5 years after implantation (Gholampour, 2018). Then, the results were compared with FSI simulation in a healthy subject. According to the results, the mean ICP domain in NCH patients were 5.3 and 2 times higher than those of healthy subjects, respectively (Gholampour et al., 2017a).

CSF pressure in NCH patients due to AS was about 5.3 times higher than that of a healthy subject. Despite the fact that after shunt implantation, CSF pressure and volume decreased significantly (pressure almost returned to the normal range of healthy subjects), the volume did not return to normal condition (with a considerable difference to the healthy subject) even 2.5 years after shunt implantation (Gholampour, 2018). In the research, compliance increase curve was studied 2.5 years after patients’ treatment. Fukuhara’s research also confirmed these results (Figure 3 b).

Reynolds number and phase difference between pressure and flow curves increased after outbreak of hydrocephalus; however, the two parameters did not demonstrate considerable decrease after shunt implantation. CSF volume and especially pressure were the most reliable parameters in evaluating the treatment methods of hydrocephalus because this parameter more than any other parameter gets closer to normal range (Gholampour et al., 2017a; Gholampour et al., 2017b; Gholampour, 2018).

Farnoush et al. (2016) studied CSF flow velocity simulation in AS and imposed pressure in the third ventricle, with and without ETV. After ETV operation, CSF flow velocity peak in Sylvius aqueduct and peak positive pressure decreased 5 and 9 times, respectively. This pressure drop was more than pressure drop and velocity reported in Gholampour research on the treatment by shunt implantation (Gholampour et al., 2017a; Gholampour, 2018).

ETV changes the time characteristics of CSF pressure waveform. After ETV operation, CSF velocity peak in Sylvius aqueduct and pressure peak reduced 2.5 and 3 times, respectively. This number is less than the similar numbers obtained from Gholampour research which suggests that shunt implantation effectiveness gets better over time. Pressure and velocity reduction in this model has been less than those of shunt implantation results in Gholampour research (Gholampour et al., 2017a).

**Figure 6.**
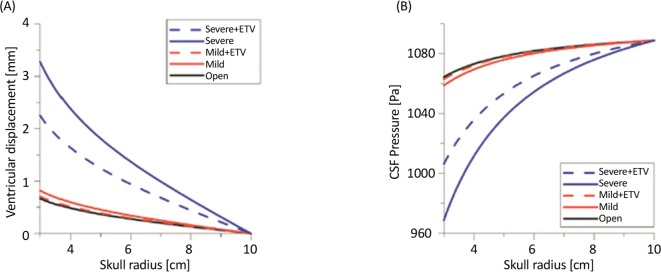
Changes in ventricular displacement and CSF pressure a. Ventricular displacement; and b. CSF pressure in open aqueductal modes, as well as in mild and severe AS cases (with and without ETV consideration) (Vardakis, Tully, & Ventikos, 2013).

Effectiveness of changes of hydrodynamic parameters of flow, including velocity and Wall Shear Stress (WSS) has been studied by Vardakis et al. during ETV treatment in open aqueductal modes as well as in mild and severe AS (Figure 6) (Vardakis et al., 2013). The results showed that maximum CSF flow velocity is about 15.6 cm/s in the healthy subjects, 45.4 cm/s in AS-related mild obstruction, and about 72.8 cm/s in a severe case of AS-related obstruction. Using ETV reduced AS velocity up to 16–17 cm/s. ETV effectiveness depends on AS level in the CSF circulation path (Farnoush et al., 2016).

## 5. Discussion

There are various methods of treating obstructive hydrocephalus, including shunt planting, ETV, and posterior fossa decompression. ETV is an alternative method of obstructive hydrocephalus treatment and effective treatment for CH patients. Of course, both ETV and shunt implantation methods improve brain compliance. Also, in infants younger than 12 months with congenital AS-related hydrocephalus, ETV is considered to be a better option. However, the method is less effective in patients with non-obstructive hydrocephalus.

All in all, there are many controversies about the efficiency of ETV and shunts in infants diagnosed with hydrocephalus. In patients younger than 19 years with PFBT-related hydrocephalus, failure occurs earlier with ETV method, compared to shunt implantation. However, after a long period of time, ETV-related complications are less than VPS-related complications. VPS is the most common method of iNPH treatment. Of course, a paucity of research has been done in relation to a higher level of ETV effectiveness in iNPH. However, results have shown that for the elderly subjects suffering from iNPH, ETV is a more effective method of treatment. Also, it is an acceptable alternative of occlusive hydrocephalus operation. Also, ETV is superior to shunt implantation in NCH patients and its effectiveness depends on AS level in CSF circulation path.

Computer simulations in which reduction of hydrodynamic parameters are compared separately for ETV and shunting implantation have shown higher effectiveness of ETV in the model with aqueductal stenosis hydrocephalus compared to shunt operation. However, in the model without aqueductal stenosis hydrocephalus, ETV was less effective than shunt implantation method. Also, the improvement of Reynolds number has been observed in AS patients after shunt operation. However, in all conditions, either before or after the treatment of patients and in healthy subjects, the Reynolds number remains within the laminar range.

Such parameters as the patient’s age, cause of hydrocephalus, and history of hydrocephalus operation are considered as important factors in the selection of appropriate treatment method (ETV or VPS). The assessment of CSF hydrodynamics showed that volume and especially pressure were the most reliable parameters in evaluating the treatment methods of hydrocephalus because this parameter has become close to normal range more than other parameters.

After ETV operation, CSF flow velocity peak in Sylvius aqueduct and peak positive pressure decreased 5 and 9 times, respectively. This pressure drop is higher than the pressure drop and velocity is seen after treatment by shunt implantation. Moreover, ETV changes time characteristics of CSF pressure waveform. After ETV operation, CSF flow velocity peak in Sylvius aqueduct and pressure peak reduce 2.5 and 3 times, respectively.

The research results on infants with hydrocephalus showed that initial treatment by ETV is more reasonable than implanting the shunt. The results also showed that both ETV and shunt implantation are appropriate options to treat pediatric hydrocephalus. In adult hydrocephalus, the initial failure in ETV occurs sooner compared to shunt therapy. However, ETV has been better during efficacy time. ETV is a potentially safer option for hydrocephalus in patients with osteoporosis. Computer simulation of hydrocephalus before and after shunt implantation and ETV treatments were showed that maximum CSF pressure is the most relevant and suitable hydrodynamic index in the analysis of these patients.
